# Biodegradable Poly(acrylic acid-*co*-acrylamide)/Poly(vinyl alcohol) Double Network Hydrogels with Tunable Mechanics and High Self-healing Performance

**DOI:** 10.3390/polym11060952

**Published:** 2019-06-01

**Authors:** Zhanxin Jing, Aixing Xu, Yan-Qiu Liang, Zhaoxia Zhang, Chuanming Yu, Pengzhi Hong, Yong Li

**Affiliations:** College of Chemistry and Environment, Guangdong Ocean University, Zhanjiang 524088, Guangdong, China; 1511331834@163.com (A.X.); zhangzx2383300@126.com (Z.Z.); yucmingdou@163.com (C.Y.); hongpengzhi@126.com (P.H.)

**Keywords:** hydrogel, double network, toughness, self-healing, biodegradable

## Abstract

We proposed a novel strategy in the fabrication of biodegradable poly(acrylic acid-*co*-acrylamide)/poly(vinyl alcohol) (P(AAc-*co*-Am)/PVA) double network (DN) hydrogels with good mechanical and self-healing properties. In the DN hydrogel system, P(AAc-*co*-Am) polymers form a network through the ionic coordinates between –COO– and Fe^3+^ and hydrogen bonding between –COOH and –CONH_2_, while another network is fabricated by the complexation between PVA and borax. The influences of the composition on the rheological behaviors and mechanical properties of the synthesized DN hydrogels were investigated. The rheological measurements revealed that the viscoelasticity and stiffness of the P(AAc-*co*-Am)/PVA DN hydrogels increase as the acrylamide and Fe^3+^ concentrations increase. At 0.05 mmol of Fe^3+^ and 50% of acrylamide, tensile strength and elongation at break of P(AAc-*co*-Am)/PVA DN hydrogels could reach 329.5 KPa and 12.9 mm/mm, respectively. These properties arise from the dynamic reversible bonds existed in the P(AAc-co-Am)/PVA DN hydrogels. These reversible bonds also give good self-healing properties, and the maximum self-healing efficiency of P(AAc-*co*-Am)/PVA DN hydrogels is up to 96.4%. The degradation test of synthesized DN hydrogels was also conducted under simulated physiological conditions and the weight loss could reach 74% in the simulated intestinal fluid. According to the results presented here, the synthesized P(AAc-*co*-Am)/PVA DN hydrogels have a potential application prospect in various biomedical fields.

## 1. Introduction

Hydrogels, which can swell and do not dissolve in water, are cross-linked polymer networks containing a large amount of water [1,2]. They are widely studied and have been applied in many fields, such as drug delivery [2,3], gene delivery [4] and tissue engineering scaffolds [5,6] due to their excellent properties, such as intelligent responsiveness, biocompatibility. However, the traditional synthetic hydrogels have generally weak mechanical strength, poor toughness and low recoverability [7]. This is largely due to their single network structure, which results in the intrinsic structure heterogeneity and the lacking effective energy dissipation mechanisms. This would greatly limit the application of hydrogel materials in tissue engineering materials and regenerative medicine that need generally to withstand large forces [8,9]. Many strategies, such as supramolecular hydrogels [10,11], nanocomposite hydrogels [12,13,14] and interpenetrating and double networks hydrogels [15,16], have been proposed to synthesize hydrogels with superior mechanical properties. Nakahata et al. [11] synthesized the supramolecular gel with extremely high toughness and elastic deformation behavior by host-guest interaction, revealing that the reversible nature of host-guest interaction gives the hydrogel self-healing property. Liu et al. [12] synthesized polyacrylamide/graphene oxide nanocomposite hydrogels by in situ free radical polymerization, found that graphene oxide nanosheets played a role of cross-linker. And the synthesized nanocomposite hydrogels showed high tensile strength, high toughness, and a large elongation at break. Liu et al. [17] developed a new design strategy to synthesize a dual ionic cross-linked polyethylene glycol (PEG)/poly(acrylamide-co-acrylic acid) (PAMAA) double network hydrogels, and found that the synthesized double network hydrogels can achieve high mechanical properties (stress of ~0.36 MPa and strain of ~1350%). Among these methods, the double-network strategy is considered as the effective route to synthesize high strength and toughness hydrogels. 

Double network (DN) hydrogels, which show outstanding comprehensive mechanical properties with respect to single network hydrogels, have two interpenetrating polymer networks [18,19]. The unique contrasting network structures and efficient energy dissipation mechanism endow the excellent mechanical properties to hydrogel materials. The introduction of the dynamically reversible network into DN hydrogel not only improves their mechanical properties, but also exhibits rapid self-healing capacity. Recently, many researchers [20,21,22] have begun to focus on the construction of double network hydrogels with self-healing property. The self-healing property of hydrogels is primary dependent on various dynamically reversible covalent, or non-covalent, such as ionic interactions [23,24] hydrogen bonding [25], hydrophobic interactions [26], host-guest interactions [27], metal-ligand interactions [28], etc. Agar/PAMAAc double network gels were synthesized by introduction of ionic coordination interaction in the second network and it was found that the double network hydrogels show extremely high mechanical properties, fast self-recovery and excellent fatigue resistance properties [29]. A double network hydrogel based on poly(vinyl alcohol) and polyacrylamide was synthesized by polymer-tannic acid multiple hydrogen bonds, found that the synthesized hydrogels have strong toughness, good self-recoverability, rapid self-healing, and versatile adhesiveness [30]. Therefore, hydrogels with excellent mechanical properties and self-healing capacity synthesized by double network technology is an important way to expand their applications.

At present, the research on the double network hydrogels with self-healing properties has focused more on this situation: one network is based on covalent bonds, the another network is based on non-covalent or reversible bonds. Double network hydrogels constructed by two reversible networks based on non-covalent bonds or reversible bonds is rarely reported. In this study, we present a simple strategy based on dynamically reversible double cross-linked networks, which can be used for the design and construction of high mechanical performances and self-healing poly(acrylic acid-co-acrylamide)/poly(vinyl alcohol) (P(AAc-*co*-Am)/PVA) double network hydrogels. Borax aqueous solution was employed to dissolve PVA, and poly(acrylic acid-co-acrylamide)/poly(vinyl alcohol) double network hydrogels were prepared via free radical polymerization in the presence of Fe^3+^. In the poly(acrylic acid-*co*-acrylamide)/poly(vinyl alcohol) double network hydrogel, borate ion would form the “di-ol” complex with PVA chains to fabricate one network, and Fe^3+^ would form the ionic coordination interactions with poly(acrylic acid-*co*-acrylamide) macromolecular chains to form another network.

## 2. Experimental Section

### 2.1. Materials and Reagents

Acrylic acid (AAc, AR), acrylamide (Am, AR) and borax (AR) were purchased from Shanghai Macklin Biochemical Co., Ltd; Polyvinyl alcohol (PVA, DP = 1750 ± 50) and Ammonium persulfate (APS, AR) were provided by the Sinopharm Chemical Reagent Co., Ltd, Shanghai, China; Ferric chloride hexahydrate (FeCl_3_⋅6H_2_O, AR) was obtained from Guangdong Guanghua Sci-Tech Co., Ltd., Shantou, Guangdong, China. All the reagents were used as received without further purification. All solutions were prepared using distilled water.

### 2.2. Synthesis of Poly(acrylic acid-co-acrylamide)/Poly(vinyl alcohol) Double Network Hydrogels

A typical synthesis process of poly(acrylic acid-*co*-acrylamide)/poly(vinyl alcohol) double network hydrogels was described as follows: Firstly, PVA (0.4 g) was added to 8 mL of H_2_O containing borax (50 mg), and stirred for 30 min at room temperature to make the swelling of PVA; Secondly, the mixture was stirred at 95 °C for 2 h to ensure the complete dissolution of PVA, and 200 μL of Fe^3+^ ions aqueous solution (0.50 mmol/mL) was added, and then cooled to 40 °C; Next, acrylic acid (1.0 g) and acrylamide (1.0 g) were added to the above solution, and 20 mg of APS was added under vigorous stirring; Subsequently, the aforementioned solution was injected to a specific mold and eliminated the bubble by vacuuming; The bubble-removed mold was placed to a thermostat at 60 °C for 6 h, and placed at −20 °C for 24 h; Eventually, the prepared sample was taken out of the mold and sealed, and denoted as Gel-8. The Fe^3+^ concentration and the ratio of AAc/Am were adjusted to synthesize poly(acrylic acid-*co*-acrylamide)/poly(vinyl alcohol) double network hydrogels with different compositions, as listed in Table 1. To meet the needs of the test, different shapes of molds are used to prepare samples with different specifications, such as cylindrical samples with diameters of 8 mm and 12 mm, films with a thickness of about 1~2 mm.

### 2.3. Hydrogel Characterization

#### 2.3.1. FT-IR 

The samples were dried in a vacuum oven to the constant weight, and ground into powder. Afterwards, a certain amount of sample was mixed with KBr, and then compressed. Eventually, the prepared discs was analyzed by Fourier transform infrared spectroscopy (FT-IR, Nicolet, Madison, WI, USA). 

#### 2.3.2. Swelling Ratio

The dried hydrogel was immersed in 80 mL of phosphate buffer solution (pH = 7.4, *I* = 0.1) at 37 °C. After a certain time interval, the sample was weighted after removing the surface water with a filter paper. The swelling ratio can be calculated by the following equation:

(1) $$swelling\;ratio\;(g/g)\;=\frac{W_{t}-W_{d}}{W_{d}}$$

where *W_d_* is the dry weight of sample, *W_t_* is the wet weight of samples after immersing in phosphate buffer solution for the time *t*.

#### 2.3.3. Rheological Characterization

The rheological properties of the synthesized hydrogels were formed on a MCR302 strain-controlled rheometer (Anton Paar, Graz, Austria) using plate-and plate geometry (diameter 25mm, gap 1000 µm), through three different modes: (a) the dynamic strain sweep from 0.01 to 500% with constant frequency of 10 rad/s was first performed at 37 °C, and the storage modulus was recorded to define the linear viscoelastic region in which the storage modulus is independent to the strain amplitude; (b) the viscoelastic parameters, including shear storage modulus, loss modulus, complex viscosity, and loss tangent as functions of angular frequency (ω) were measured over the ω range of 0.1–100 rad/s at strain= 1%, and the measured temperature was controlled at 37 °C; (c) the alternate step strain sweep test was performed at a fixed angular frequency (10 rad/s) at 37 °C, amplitude oscillatory strains were switched from small strain (γ = 1.0%) to subsequent large strain (γ=100%) with 100 s for every strain interval. The storage modulus, loss modulus dependence of time was recorded. 

#### 2.3.4. Tensile Testing

Tensile test of all samples was measured using a 500N universal testing machine (Jinan Zhongluchang Testing Machine Manufacturing Co., Ltd, Jinan, Shandong, China) at a uniaxial stretching speed of 50 mm/min at the room temperature. The size of cylindrical sample was 8 mm in diameter and 60 mm in length. Each sample was repeated five times to ensure repeatability.

#### 2.3.5. Compression Testing

Compression experiment was performed on a 10KN universal testing machine (Jinan Zhongluchang Testing Machine Manufacturing Co., Ltd, Jinan, Shandong, China). The cylindrical samples of 12 × 20 mm^2^ (diameter×height) were placed on the lower plate and compressed by the upper plate at a compression speed of 20 mm/min at the room temperature. The tests of all samples were conducted in triplicate.

#### 2.3.6. Self-Healing Property

Self-healing property of the prepared hydrogel was evaluated by comparing the tensile fracture behaviors of original and healed samples. The cylindrical samples (8 × 60 mm^2^) were first cut in the center, then the two halves were contacted together and put in a plastic syringe with a diameter of 8 mm. The plunger of the syringe was pressed to ensure full contact with the interface. The healing time was varied from 8 h to 48 h, and the healing temperature was varied from 30 °C to 50 °C. After self-healing, tensile tests of the healed samples were measured to evaluate the healing efficiency. The healing efficiency (*HE_stress_*, *HE_strain_*) can be calculated by the followed equation:

(2) $$HE_{stress}(\%)=\frac{F_{h}}{F_{o}}\times 100\%$$

(3) $$HE_{strain}(\%)=\frac{S_{h}}{S_{o}}\times 100\%$$

where *F_o_ and S_o_* are the tensile strength and elongation at break of original sample, respectively; *F_h_* and *S_h_* are the tensile strength and elongation at break of the healed samples, respectively. The average values and errors were calculated from at least three independent samples for each specimen.

#### 2.3.7. Degradation Testing

Degradation experiments were performed in phosphate buffer solution (pH = 7.4, *I* = 0.1) and in simulated intestinal fluid with 50 U/mL trypsin (pH = 7.4, *I* = 0.1). The dried cylindrical hydrogels (about 0.15 g) were first swollen in phosphate buffer solution for 48 h. Then, the swollen hydrogels were placed in 80 mL of degradation fluid and degraded in thermostatic oscillator (30 r/min) at 37 °C for 10 days. The degradation ratio was determined by the following equation:

(4) $$weight\;loss\;(\%)=\frac{W_{o}-W}{W_{o}}\times 100\%$$

where $W_{o}$ and *W* are the dried weight of samples before and after degradation, respectively. The tests of all samples were conducted in triplicate.

## 3. Results and Discussion

### 3.1. Formation and Structure Analysis

**Scheme 1:**
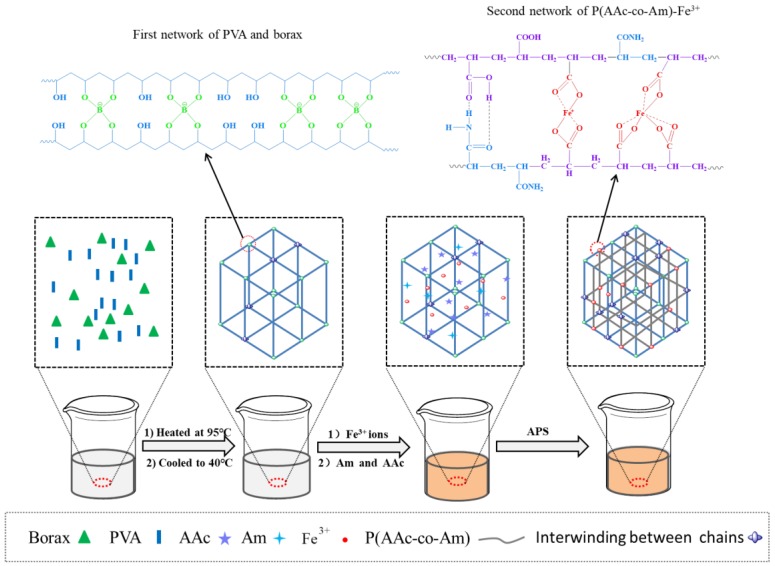
Schematic diagram of synthesis of P(AAc-co-Am)/PVA double network hydrogels.

The formation mechanism of poly(acrylic acid-*co*-acrylamide)/poly(vinyl alcohol) double network hydrogels are displayed in Scheme 1. Firstly, PVA was dissolved in the borax aqueous solution at 95 °C. During the experiment, we found that the fluidity of the solution was getting worse and the white analogous hydrogel formed as the temperature decreased. This is attributed to the formation of a network structure of PVA and borax, which is a reversible crosslinking reaction. The crosslinking mechanism of borate ion with PVA chains was known to be a “di-ol” complex, which was formed between one borate ion and two di-ol units [31,32]. When the temperature was cooled to 40 °C, Fe^3+^ ions aqueous solution is added. The phenomenon reveals that the incorporation of Fe^3+^ ions improves the fluidity of the mixture, indicating that the part of crosslinking points of the borate ion with PVA chains is damaged. This also demonstrates that the formed bond of the borate ion with PVA chains is reversible. Next, acrylic acid and acrylamide were added, and the APS aqueous solution was added to initiate the polymerization of AAc and Am monomers. This would form the linear poly(acrylic acid-*co*-acrylamide) macromolecular chain with pendant carboxyl groups and amide groups. The coordination between Fe^3+^ ions and carboxyl groups could play a role of cross-linking point, resulting in the formation of ionic cross-linked network based on poly(acrylic acid-*co*-acrylamide). While the hydrogen bonding force may be also form between the pendant carboxyl group and amide group. Therefore, a double network hydrogel based on PVA and poly(acrylic acid-*co*-acrylamide) was synthesized. In the double network structure, the first network based PVA and borax and the second network based poly(acrylic acid-*co*-acrylamide) and Fe^3+^ ions are dynamically reversible. These structures endow the poly(acrylic acid-*co*-acrylamide)/poly(vinyl alcohol) double network hydrogels with excellent mechanical strength and self-healing properties.

**Figure 1:**
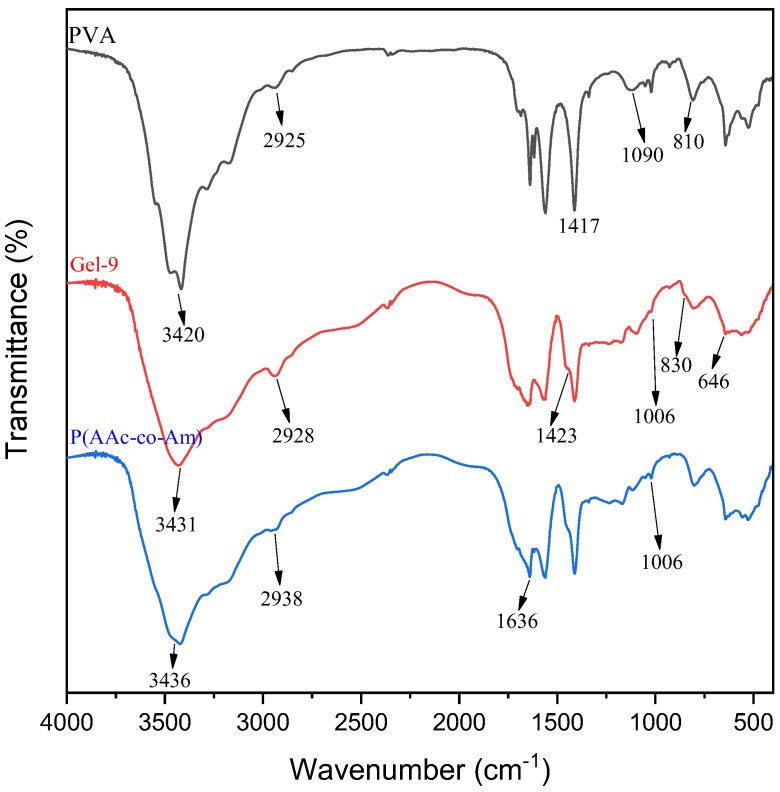
Fourier transform infrared spectroscopy (FT-IR) spectra of PVA, P(AAc-*co*-Am) and P(AAc-*co*-Am)/PVA double network hydrogel (Gel-9).

To further analyze the structure of poly(acrylic acid-*co*-acrylamide)/poly(vinyl alcohol) double network hydrogels, FT-IR spectra of PVA, poly(acrylic acid-*co*-acrylamide), poly(acrylic-*co*-acrylamide)/poly(vinyl alcohol) double network hydrogel were also measured, as displayed in Figure 1. In the spectrum of PVA, the broad peak at about 3420 cm^−1^ is assigned to –OH and C–OH stretching. The peak at 2925 cm^−1^ is attributed to asymmetric –CH_2_- group stretching vibration. The peak at 1417 cm^−1^ is due to -OH bending vibration of the hydroxyl group. The peaks at 1090 and 810 cm^−1^ are ascribed to C–O stretching and C–C stretching vibration, respectively [33]. For poly(acrylic acid-co-acrylamide), the peak at 3436 cm^−1^ is attributed to the -NH stretching vibration of the acrylamide unit, which overlapped with the -OH groups of acrylate units. The peak at 2938 cm^−1^ are assigned to the C–H absorption band caused by the methyl and methylene groups of poly(acrylic acid-co-acrylamide). It can be observed that the superposition of amide group (1650 cm^−1^) and C=O in carboxyl group (1720 cm^−1^) result in the band shift of the C=O stretching vibration (1636 cm^−1^), suggesting that the strong hydrogen bonds are formed between –COOH and –CONH_2_ [34,35]. In FT-IR spectrum of sample Gel-9, there presents several characteristic peaks of poly(acrylic acid-*co*-acrylamide)/poly(vinyl alcohol) double network hydrogels. These peaks at 1423 cm^−1^, 828 cm^−1^, 650 cm^−1^ are ascribed to asymmetric stretching relaxation of B-O-C, B-O stretching from residual B(OH)_4_, bending of B-O-B linkages within borate networks, respectively [36,37]. This reveals that the polyvinyl alcohol-based dynamically reversible cross-linking network is formed due to the occurrence of complexation between PVA and borate. The reduced absorption peak at 1006 cm^−1^ is also observed, assigned to the formation of coordination interactions between Fe^3+^ and –COO^−^ groups in the poly(acrylic acid-*co*-acrylamide) chains [23]. This indicates that another physically cross-linking network is formed at between Fe^3+^ and poly(acrylic acid-co-acrylamide). These results demonstrate that poly(acrylic acid-*co*-acrylamide)/poly(vinyl alcohol) double network hydrogels are successfully synthesized by dynamically reversible cross-linking network. 

**Figure 2:**
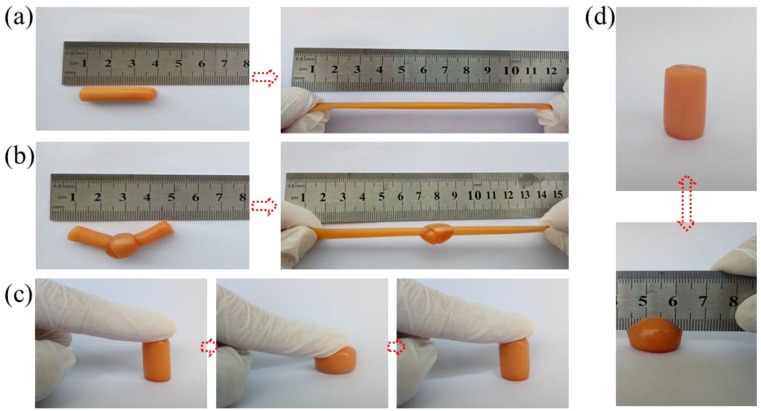
Photographs of P(AAc-*co*-Am)/PVA double network hydrogels (Gel-9) under different conditions demonstrating their excellent mechanical deformation and recoverability. The hydrogels showing their ability to withstand stretching (**a**), knotting (**b**), compression (**c**,**d**).

Poly(acrylic acid-*co*-acrylamide)/poly(vinyl alcohol) double network hydrogels exhibit excellent mechanical properties that can withstand various deformations, as shown in Figure 2. It can be observed that the cylindrical sample could be easily stretched to 300% of their original length without breaking, and the knotted sample could be stretched (as Figure 2a,b). The cylindrical sample could self-recover to its origin height after removing the applied force, as in Figure 2c. We also found that the hydrogel could recover its original shape without any damage under a sharp blade compression. These phenomena indicate that the synthesized hydrogels possess outstanding toughness and self-recovery properties. Hydrogels are hydrophilic materials composed of water and polymer networks, so their mechanical properties are directly related to water content [31]. One of the important applications of hydrogel materials is tissue engineering. However, for human tissue, its water content is about 70%. Therefore, in this study, the water content of the synthesized P(AAc-*co*-Am)/PVA double network hydrogels is controlled at ~70% (as listed in Table 1).

### 3.2. Swelling Behavior of Poly(acrylic acid-co-acrylamide)/Poly(vinyl alcohol) Double Network Hydrogels

**Figure 3:**
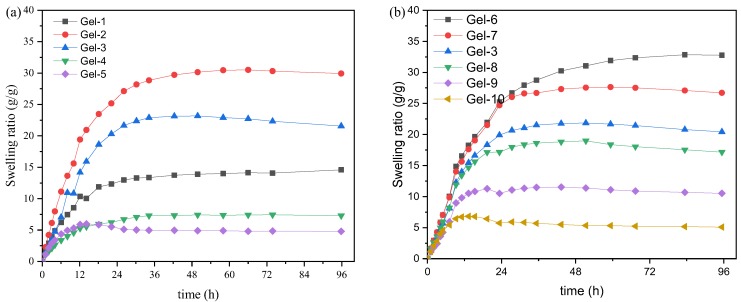
Variation of swelling ratio of P(AAc-co-Am)/PVA double network hydrogels as function of time at 37 °C: (**a**) hydrogels with various Am/AAc ratios; (**b**) hydrogels with various Fe^3+^ concentrations.

Swelling behaviors of the synthesized double network hydrogels were also investigated in phosphate buffer solution (pH = 7.4, *I* = 0.1) at 37 °C, and the swelling ratios as function of time are shown in Figure 3. It is clear that all samples can reach the equilibrium swollen state after immersing for 72 h, and the swelling ratio is closely related to the hydrogel composition. As in Figure 3a, the swelling ratio first increases and then decreases with increasing of the content of Am. Swelling ratio of hydrogel with 30% of Am reaches the maximum (about 30 g/g). This is because a non-ionic hydrophilic groups (–CONH_2_) is introduced into the gel network, and the -CONH_2_ has a low ionization degree in a near neutral environment [38]. This would result in a certain resistance of the gel to salty in the buffer solution. While an increase in the content of Am causes a decrease in the AAc content, resulting in a decrease in the electrostatic repulsion caused by –COO^−^ groups [39]. The influence of the Fe^3+^ concentrations on swelling ratios is displayed in Figure 3b. With increasing of the Fe^3+^ concentrations, the swelling ratio obviously decreases, and the time required to reach the swelling equilibrium becomes shorter. This is mainly attributed to the cross-linking of Fe^3+^. The increase Fe^3+^ concentration leads to the formation of more coordination interactions, which restrain the swelling capacity of hydrogels. And the coordination interactions between Fe^3+^ and –COO^−^ groups consume carboxyl anions, which weakens the electrostatic repulsion between the polymer segments. This is another reason for the decrease in swelling ratios of PVA/poly(acrylic acid-*co*-acrylamide) double network hydrogels. A similar result has been reported by in the system of PAAc-Fe^3+^/CNC hydrogels [16].

### 3.3. Rheological Behaviors of Poly(acrylic acid-co-acrylamide)/Poly(vinyl alcohol) Double Network Hydrogels

**Figure 4:**
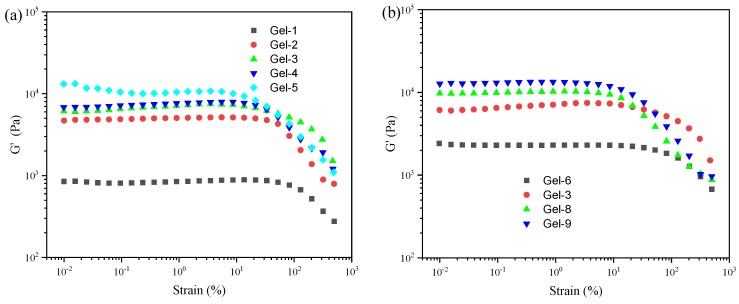
The strain amplitude sweep test of P(AAc-*co*-Am)/PVA double network hydrogels at a fixed angular frequency (10 rad/s) at 37 °C: (**a**) hydrogels with various Am/AAc ratios; (**b**) hydrogels with various Fe^3+^ concentrations.

The storage modulus (G′) as function of strain for poly(acrylic acid-*co*-acrylamide)/poly(vinyl alcohol) double network hydrogels are shown in Figure 4. It is clear that all samples displayed a typical elastic response [16,18]. The G′ of DN hydrogels are independent of the applied strain when the strain is lower than the certain value (γ_c_), revealing a viscoelastic solid. The G′ values gradually decrease above the certain value. This indicates that DN hydrogels undergoes a transition from the quasi-solid state to a quasi-liquid state. As shown in Figure 4a, with increasing of the Am contents, the G′ of DN hydrogels increases, while the corresponding γ_c_ decreases. This indicated that the increased Am contents improve the stiffness, but reduce the ability to sustain larger deformation [31]. It can be observed in Figure 4b that the G′ of DN hydrogel increases as the Fe^3+^ concentrations increases, but the corresponding γ_c_ decreases. This reveals that the increased Fe^3+^ concentrations increase the stiffness of the DN hydrogels, leading to the decreasing of the ability to maintain larger deformation. This is attributed to the cross-linking density increased by ionic coordinates between Fe^3+^ and –COO^−^. To ensure that the dynamic oscillatory deformation was a linear viscoelastic region, a strain (γ = 1%) was selected in the following oscillation tests.

**Figure 5:**
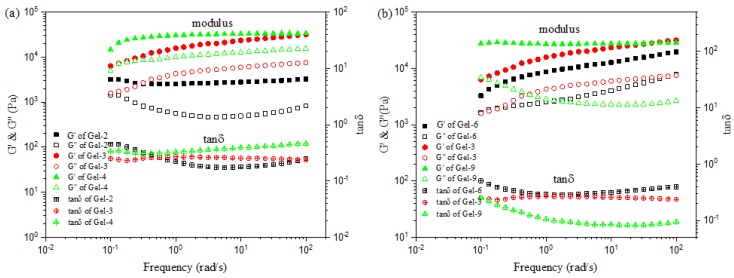
Variation of storage modulus (G′) and loss modulus (G′′) as function of frequency for samples measured at γ = 1%: (**a**) hydrogels with various AAc/Am ratios; (**b**) hydrogels with various Fe^3+^ concentrations.

To further explain the influence of the composition on the viscoelastic properties of P(AAc-*co*-AM)/PVA DN hydrogels, oscillatory measurements were performed at 37 °C, as shown in Figure 5. It is clear that the storage modulus (G′) is larger than the loss modulus (G′′) under all frequency range, and the moduli are significantly weak dependent on the frequency. This is a characteristic of a solid-like viscoelastic behavior [40,41]. It is well known that the storage modulus can be used to evaluate the extent of gel network formation. The higher storage modulus of the gel, the stronger is gel intensity [42]. As in Figure 5a, when increasing the Am content, the G′ and G′′ obviously increases. The possible reason for this is that the increased Am content leads to the formation of hydrogen bonding between –COOH and –CONH_2_, improving the cross-linking points in the poly(acrylic acid-*co*-acrylamide) network. It is also observed that Tanδ = G′′/G′ blow 0.40 reveals weak dependency on the frequency. Figure 5b shows the G′ and G′′ of hydrogels with different Fe^3+^ concentration as a function of frequency. It can be found that the G′ of hydrogels increases as the Fe^3+^ concentration increases, while the G′′ first increases and then decreases. Tanδ = G′′/G′ below 0.45 shows also weak dependency on the frequency. A similar phenomenon has also been reported by Hussain et al in the hydroxyethyl cellulose/P(AAc-*co*-Am)-Fe^3+^ hydrogels [43]. 

**Figure 6:**
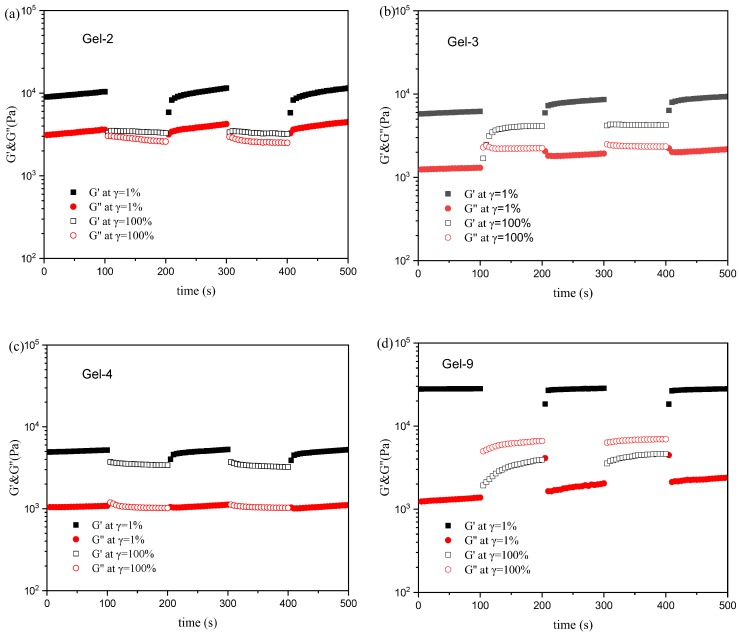
Alternate step strain test with small strain to subsequent large strain with 100 s for every strain interval at fixed angular frequency (10 rad/s) at 37 °C: (**a**) Gel-2; (**b**) Gel-3; (**c**) Gel-4; (**d**) Gel-9

Figure 6 shows the G′ and G′′ dependence of time in continuous step strain measurements for DN hydrogels. As Figure 6a, when a small-amplitude oscillatory force (γ = 1%) was applied, the G′ and G′′ of Gel-2 are about 10.0 and 3.5 KPa, respectively. Under the application of a large-amplitude oscillatory force (γ = 100%), the G′ and G′′ of Gel-2 decrease to 3.4 KPa and 2.5 KPa, respectively. However, the G′ and G′′ of Ge-2 could recover the initial values when amplitude oscillatory force is decreased once again to 1.0%. It is clear that loss tangent at γ = 1% and γ = 100% are about 0.35, and 0.80, respectively, indicating that sample Gel-2 always presented a solid nature when the amplitude oscillatory alternated between 1% and 100%. It can be found by comparing Figure 6a–c that G′ and G′′ of P(AAc-*co*-Am)/PVA DN hydrogels decreases under the same oscillatory force as the content of Am increases. The tanδ at γ = 100% decreases as the Am content increase. In Figure 6d, it is clear that the tanδ of Gel-9 with 0.20 mmol Fe^3+^ at γ = 1% and γ = 100% are 0.05~0.08 and 1.5~2.0, respectively, indicating that the sample undergoes a transition from the original quasi-solid state to the quasi-liquid state. These results indicate that the P(AAc-*co*-Am)/PVA DN hydrogels show good toughness and self-recover properties.

### 3.4. Mechanical Properties of Poly(acrylic acid-co-acrylamide)/Poly(vinyl alcohol) Double Network Hydrogels

**Figure 7:**
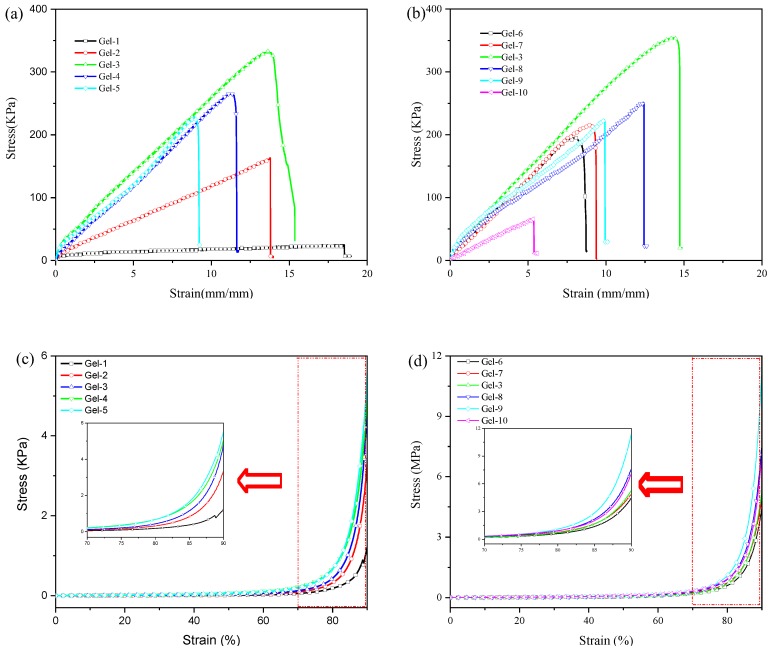
Tensile stress-strain curves (**a,b**) and compressive stress-strain curves (**c,d**) of P(AAc-co-Am)/PVA double network hydrogels: (**a,c**) hydrogels with various Am/AAc ratios; (**b,d**) hydrogels with various Fe^3+^ concentrations.

Mechanical properties of poly(acrylic acid-*co*-acrylamide)/poly(vinyl alcohol) double network hydrogels were investigated by tensile and compression tests. The obtained curves are displayed in Figure 7, and tensile strength, elongation at break and compressive strength obtained from Figure 7 are listed in Table 2. Figure 7a displays the typical tensile stress-strain curves of hydrogels with various Am/AAc ratios. For sample Gel-1 with 10% of Am content, the tensile strength and elongation at break are 24.9 KPa and 19.5 mm/mm, respectively. By increasing the Am contents, tensile strength of DN hydrogels first increases and then decreases, while the elongation at break is always decreasing. The tensile strength of P(AAc-*co*-Am)/PVA DN hydrogel with 50% of Am content is maximum (329.5 KPa), and its elongation at break is 12.9 mm/mm. By increasing the Am content, the network based on poly(AAc-*co*-Am)-Fe^3+^ increases the hydrogen bonding between –COOH and –CONH_2_ at the certain Fe^3+^ concentrations, resulting in the increase of reversible cross-linking points, which would enhance the mechanical properties of DN hydrogels. By further increasing the Am contents, the hydrogen bonding dominates the poly(AAc-*co*-Am)-Fe^3+^. The hydrogen bonding is weak with respect to the ionic coordinates between -COO^−^ and Fe^3+^. An increase in the content of Am could lead to the Fe^3+^ surplus, resulting in that the ionic coordinates existed between Fe^3+^ and –COO- group are more likely as bidentate coordinates and/or even monodentate coordinates [44]. The typical compressive stress-strain of hydrogels with various Am/AAc ratios are displayed in Figure 7c. By increasing the Am content, the compressive strength increases. For P(AAc-*co*-Am)/PVA DN hydrogels with 50% of Am, its compressive strength at strain 90% could reach 5.7 MPa. This is consistent with the results of rheology.

The typical tensile stress-strain curves of P(AAc-*co*-Am)/PVA DN hydrogels with various Fe^3+^ concentrations are displayed in Figure 7b. It can be observed that the tensile strength and elongation at break first increase and then decrease as the Fe^3+^ concentrations increase, and P(AAc-*co*-Am)/PVA DN hydrogel with 0.05 mmol Fe^3+^ presents the most tensile strength. For DN hydrogels with lower Fe^3+^ concentrations, the dynamic reversible coordination bonds allow the material to break and re-form during stretching. This is because the ionic coordinates between Fe^3+^ and –COO^−^ groups is mainly in the form triple-dentate coordinates; when the Fe^3+^ concentration exceeds the certain value, the triple-dentate coordinates in the DN hydrogels decreases. The ionic coordinates between –COO^−^ and Fe^3+^ are more likely as bidentate coordinates and/or even monodentate coordinates, which could decrease in the lower cross-linking density of DN hydrogels. The high Fe^3+^ concentration could occur the negative effect for the free radical copolymerization reaction of AAc and Am monomers, which is because Fe^3+^ may consume active free radicals [17,45]. The typical compressive stress-strain curves of hydrogels with various Fe^3+^ concentrations are shown in Figure 7d. It is observed that the compressive strength of DN hydrogels first increases and then decreases as the Fe^3+^ concentrations increase, which shows similar trend with tensile test of DN hydrogel with various Fe^3+^ concentrations. The compressive strength of P(AAc-*co*-Am)/PVA DN hydrogel with 0.20mmol Fe^3+^ is the maximum and could reach 11.4 MPa at strain 90%.

The hysteresis behavior and self-recovery properties of P(AAc-*co*-Am)/PVA DN hydrogels were also evaluated, as displayed in Figure S1. It is clear that the loading-unloading curves of P(AAc-*co*-Am)/PVA DN hydrogels (Gel-8) under various strains show obvious hysteresis loops, revealing that the synthesized DN hydrogels could dissipate a large amount of energy in cyclic test. The area of loop increases as the strain increases, showing a higher energy dissipation. This may be attributed to the non-covalent interaction and reversible covalent. A similar result has been reported by Shao et al. [16] in the physically cross-linked PAA-CNF-Fe^3+^ hydrogels. It can be also observed from Figure S1b that the stress-strain curves gradually recover to the original loading pathway as the resting time increases, especially for the lower strain (<300%). This reveals that self-recovery properties of P(AAc-*co*-Am)/PVA DN hydrogels show time-dependence. This is assigned to the reorganizing of the reformed bonds. In this process, the ionic coordinates, “di-ol” complex and hydrogen bond act as sacrificial bonds, which endows P(AAc-*co*-Am)/PVA DN hydrogels with good toughness and self-recovery.

### 3.5. Self-Healing Properties and Self-Healing Mechanism of Poly(acrylic acid-co-acrylamide)/Poly(vinyl alcohol) Double Network Hydrogels

**Figure 8:**
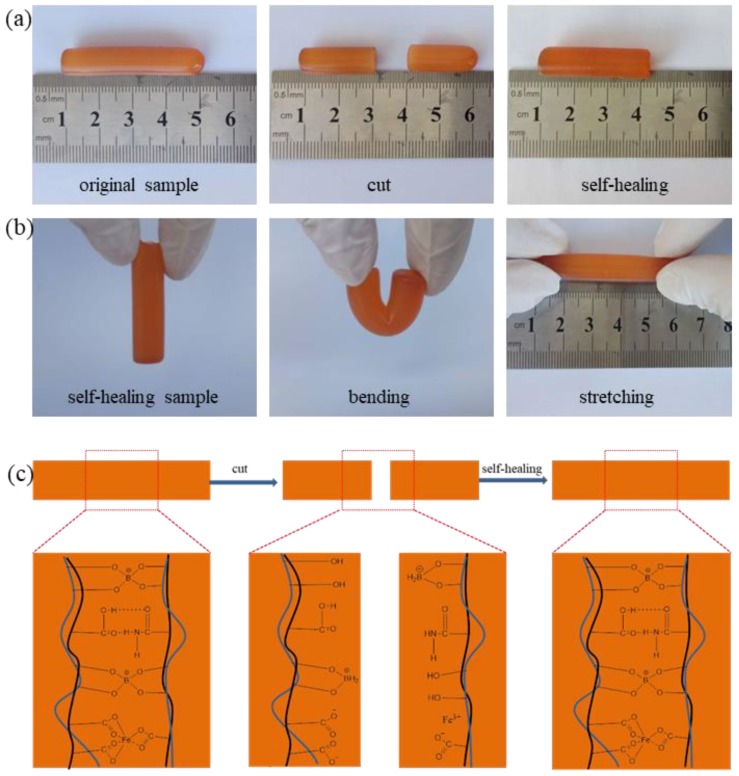
(**a**,**b**) presenting the macroscopic self-healing behavior of P(AAc-*co*-Am)/PVA double network hydrogels (Gel-9); (**c**) Schematic of the self-healing mechanism of P(AAc-*co*-Am)/PVA double network hydrogels.

During the experiment, we found that the prepared poly(acrylic acid-*co*-acrylamide)/poly(vinyl alcohol) double network hydrogels not only present highly stretchable and resilient properties, but also show the certain self-healing capacity. As shown in Figure 8a, a cylindrical sample was cut into two sections. When the freshly fractured surfaces contact, two sections can merge to form a single, cylindrical sample. The self-healed samples can undergo significant deformation, as displayed in Figure 8b. These phenomena revealed that the synthesized P(AAc-*co*-Am)/PVA double network hydrogels presents good self-healing performance, which is mainly assigned to the special chemical structures. As shown in Figure 8c, the self-healing capacity of P(AAc-*co*-Am)/PVA double network hydrogels are mainly due to complexation reaction between borate ion and two di-ol units in PVA chains, electrostatic interaction between carboxyl anion and Fe^3+^ and hydrogen bonding between carboxyl and amide groups, which are dynamically reversible bonds [46,47,48,49]. When samples were cut in half, these bonds were damaged. However, the halves were in contact, the carboxyl anion and Fe^3+^ could produce electrostatic interaction, accelerating the integration of two halves. Simultaneously, complexation reaction between borate ion and two di-ol units in PVA chains and hydrogen bonding between carboxyl and amide in the inter-interface formed again. These reversible bonds together maintain and improve the self-healing capacity of poly(acrylic acid-*co*-acrylamide)/poly(vinyl alcohol) double network hydrogels. 

**Figure 9:**
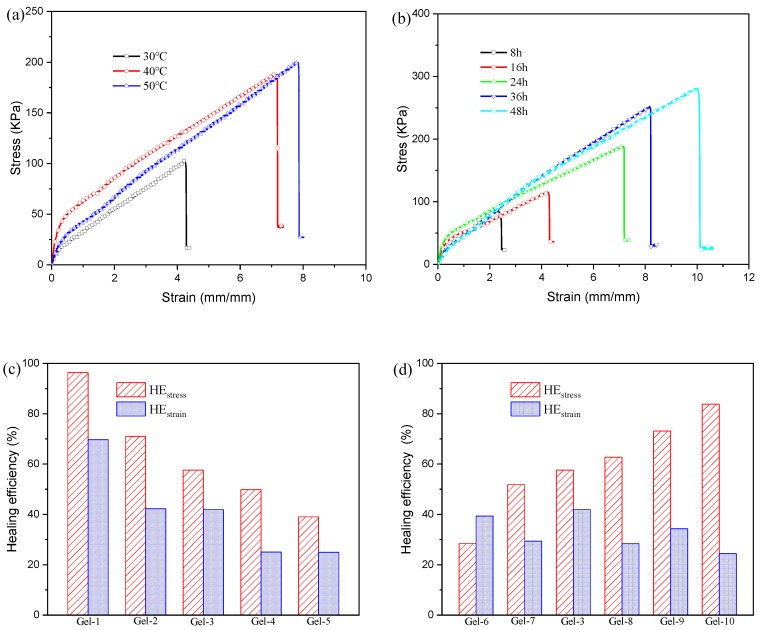
Tensile stress-strain curves (**a,b**) of healed sample Gel-3: (**a**) various self-healing temperatures; (**b**) various self-healing times; Healing efficiency (**c,d**): (**c**) hydrogels with different Am/AAc ratios; (**d**) hydrogels with different Fe^3+^ concentrations.

The self-healing conditions of poly(acrylic acid-*co*-acrylamide)/poly(vinyl alcohol) double network hydrogels were further discussed and analyzed, and the results were displayed in Figure 9a,b) As Figure 9a, the tensile strength and elongation at break of sample healed at 30 °C are 100.8 KPa and 4.2 mm/mm, respectively. By increasing the self-healing temperature, the tensile strength and elongation at break obviously increases. The influence of self-healing time on the self-healing capacity was shown in Figure 9b. The tensile strength and elongation at break increase as the self-healing time increases, and the tensile strength and elongation at break at 48 h could reach 281.0 KPa and 10.0 mm/mm, respectively. Yuan et al [24] also reported that the increase time and temperature contributed to the improvement of self-healing ability, which may be attributed to the enhanced mobility of the polymer chains at longer times and higher temperatures. To deeply analyze the self-healing properties of P(AAc-*co*-Am)/PVA double network hydrogels, the self-healing efficiency was calculated by the ratio of the tensile strength of the self-healing sample to the tensile strength of the original sample. It can be known that the sample Gel-3 at 40 °C for 48 h could recover to 85.3% of the original tensile strength. There results reveal that the self-healing properties of the synthesized DN hydrogels improve as the self-healing temperature and time increase. Therefore, the DN hydrogels exhibit excellent self-healing ability. Furthermore, the effect of the compositions on the self-healing property was discussed at a temperature 40 °C for 24 h. Tensile strength and elongation at break of healed samples are listed in Table 2, and the obtained healing efficiency is shown in Figure 9c,d. As in Figure 9c the *HE_stres_*_s_ and *HE_strain_* of DN hydrogel with 10% of Am content are 96.4% and 69.7%, respectively. By increasing the Am contents, the self-healing efficiency rapidly decreases. For DN hydrogel with 90% of Am content, its *HE_stres_*_s_ and *HE_strain_* are only 39.0% and 25.1%, respectively. Figure 9d shows the influence of the Fe^3+^ concentrations on self-healing efficiency. It is clear that the *HE_stres_*_s_ increases from 28.4% to 83.8% as the Fe^3+^ concentration increases from 0.01 mmol to 0.30 mmol. 

### 3.6. Biodegradation Properties of Poly(acrylic acid-co-acrylamide)/Poly(vinyl alcohol) Double Network Hydrogels

**Figure 10:**
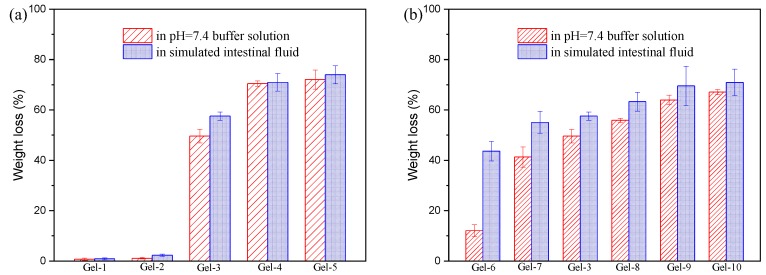
The weight loss of P(AAc-*co*-Am)/PVA double network hydrogels after 10 days of degradation in phosphate buffer solution (pH = 7.4, *I* = 0.1) and the simulated intestinal fluid: (**a**) hydrogels with various Am/AAc ratios; (**b**) hydrogels with various Fe^3+^ concentrations.

The biodegradability of hydrogels has an important influences on applications in biomedicine and tissue engineering. Therefore, the degradation properties of P(AAc-*co*-Am)/PVA DN hydrogels were also investigated, and the obtained results are displayed in Figure 10. As in Figure 10, the weight loss in phosphate buffer solution (pH = 7.4, *I* = 0.1) is significantly less than that of the simulated intestinal fluid. The simulated intestinal fluid contains trypsin compared to the phosphate buffer solution, suggesting that trypsin promotes the degradation of P(AAc-*co*-Am)/PVA DN hydrogels. However, the mechanism of the influence of trypsin on the degradability of P(AAc-*co*-Am)/PVA) double network hydrogels is still unclear, and it needs further research. Figure 10a shows the weight loss after the degradation of P(AAc-*co*-Am)/PVA DN hydrogels with various Am/AAc ratios. It is clear that the weight loss of Gel-1 and Gel-2 is less than 3%, which may be related to the high AAc content. Sun et al [50] reported the analogical result that the PAAc hydrogel is difficult to degrade by buffer solution and enzymes. By increasing the Am content, the weight loss significantly increases. For DN hydrogel with 90% of Am content, the weight loss in phosphate buffer solution and simulated intestinal fluid could reach 70% and 74%, respectively. Figure 10b displayed the influences of Fe^3+^ concentrations on degradation properties of P(AAc-*co*-Am)/PVA) DN hydrogels. By increasing the Fe^3+^ concentrations, the weight loss is enhanced. For P(AAc-*co*-Am)/PVA) DN hydrogels with 0.30 mmol Fe^3+^, the weight loss in phosphate buffer solution and simulated intestinal fluid are 66% and 71%, respectively. It can be also found from Figure 10b that the weight loss of the sample with lower Fe^3+^ concentrations in the simulated intestinal fluid is much larger than that of phosphate buffer solution, and the difference decreases as the Fe^3+^ concentration increases. This is because the network based on poly(acrylic acid-*co*-acrylamide) and Fe^3+^ not only exists the ionic coordination between –COO– and Fe^3+^, but has the hydrogen bonding between –COOH and –CONH_2_. The hydrogen bond is weak with respect ionic coordination. This result reveals that trypsin at the simulated intestinal fluid has different ability to degrade ionic coordination and hydrogen bond.

## 4. Conclusions

We synthesized a novel biodegradable poly(acrylic acid-*co*-acrylamide)/poly(vinyl alcohol) double network hydrogels with good mechanical properties and efficient self-healing properties on the basis of dynamically reversible bonds. The properties of P(AAc-*co*-Am)/PVA double network hydrogels, such as swelling ratios, mechanical properties, self-healing properties and degradability, are adjusted by tuning the composition. The incorporation of reversible cross-linking network could provide the P(AAc-*co*-Am)/PVA double network hydrogels with high extensibility, and self-recovery ability. P(AAc-*co*-Am)/PVA double network hydrogels show the tensile strength of 329.5 KPa and elongation at break of 12.9 mm/mm, respectively. The compressive strength could reach 11.4 MPa at strain 90%. What is more, the incorporation of dynamically reversible bonds endow the hydrogel with the excellent self-healing properties and degradability, and the self-healing efficiency and weight loss of P(AAc-*co*-Am)/PVA double network hydrogels could also reach 96.4% and 74%, respectively. Therefore, the P(AAc-*co*-Am)/PVA double network hydrogels would have a great potential application in various biomedical fields.

## Figures and Tables

**Table 1:** P(AAc-*co*-Am)/PVA double network hydrogels with various compositions.

Samples	Am/AAc (g/g)	Fe^3+^ (mmol)	APS (g)	PVA (g)	Borax (mg)	H_2_O (mL)	Water content (wt %) *^a^*
Gel-1	1:9	0.05	0.02	0.4	50	8	70.51 ± 0.74
Gel-2	3:7	0.05	0.02	0.4	50	8	71.74 ± 0.02
Gel-3	5:5	0.05	0.02	0.4	50	8	69.44 ± 0.73
Gel-4	7:3	0.05	0.02	0.4	50	8	73.45 ± 0.50
Gel-5	9:1	0.05	0.02	0.4	50	8	71.60 ± 0.44
Gel-6	5:5	0.01	0.02	0.4	50	8	70.17 ± 0.59
Gel-7	5:5	0.025	0.02	0.4	50	8	72.39 ± 0.82
Gel-8	5:5	0.10	0.02	0.4	50	8	72.75 ± 0.54
Gel-9	5:5	0.20	0.02	0.4	50	8	71.26 ± 0.63
Gel-10	5:5	0.30	0.02	0.4	50	8	75.75 ± 0.22

*^a^* Water content of the hydrogels were calculated by the following equation: $water\;content(\%)=\frac{w_{o}-w_{d}}{w_{d}}\times 100\%$, where $w_{o}$ and $w_{d}$ are the weight of the hydrogel before and after drying.

**Table 2:** Mechanical properties of P(AAc-*co*-Am)/PVA double network hydrogels.

Samples	Original			Healed *^b^*	
	Tensile Strength (KPa)	Elongation at Break (mm/mm)	Compressive Strength *^a^* (MPa)	Tensile Strength (KPa)	Elongation at Break (mm/mm)
**Gel-1**	24.9 ± 2.3	19.5 ± 1.3	1.0 ± 0.2	24.0 ± 6.3	13.6 ± 2.0
**Gel-2**	152.5 ± 16.6	14.7 ± 3.4	2.8 ± 0.3	108.3 ± 22.5	6.2 ± 1.8
**Gel-3**	329.5 ± 30.6	12.9 ± 2.8	4.9 ± 0.5	189.8 ± 10.4	5.4 ± 1.3
**Gel-4**	255.0 ± 21.8	10.8 ± 0.8	5.1 ± 0.4	127 ± 16.9	2.71 ± 0.3
**Gel-5**	213.5 ± 16.4	8.8 ± 1.0	5.7 ± 0.6	83.2 ± 15.6	2.21 ± 0.3
**Gel-6**	196.5 ± 25.5	7.6 ± 1.3	4.6 ± 0.4	55.9 ± 9.1	2.2 ± 0.2
**Gel-7**	224.2 ± 19.8	9.9 ± 0.4	4.8 ± 0.6	116.1 ± 22.9	2.9 ± 0.8
**Gel-8**	246.8 ± 22.8	11.6 ± 0.9	7.0 ± 0.7	154.8 ± 5.2	3.3 ± 0.2
**Gel-9**	210.0 ± 27.9	10.3 ± 1.1	11.4 ± 1.0	153.4 ± 11.2	3.53 ± 0.6
**Gel-10**	64.9 ± 6.0	4.9 ± 0.5	6.8 ± 0.8	54.4 ± 7.2	1.2 ± 0.2

*^a^* Compressive strength at 90% strain; *^b^* self-healing condition at 40 °C for 24h.

## Supplementary Materials

The following are available online at https://www.mdpi.com/2073-4360/11/6/952/s1. Figure S1: Hysteresis and self-recovery properties of P(AAc-co-AM)/PVA DN hydrogels at room temperature: (a) cyclic tensile loading-unloading curves under different strains at certain resting time (5 min) between two successive measurements; (b) cyclic tensile loading-unloading curves under different strains at certain resting time (30 min) between two successive measurements.
