# Supplementary material for: Biodegradable Poly(acrylic acid-co-acrylamide)/Poly(vinyl alcohol) Double Network Hydrogels with Tunable Mechanics and High Self-healing Performance
Source: Polymers (Basel). 2019 Jun 1;11(6):952. doi: 10.3390/polym11060952 (PMC6631433; doi:10.3390/polym11060952)
Supplement: Supplementary file 1 [file polymers-11-00952-s001.pdf]

Supporting Information

Figure S1 Hysteresis and self-recovery properties of P(AAc-co-AM)/PVA DN hydrogels at room temperature: (a) cyclic tensile loading-unloading curves under different strains at certain resting time (5min) between two successive measurements; (b) cyclic tensile loading-unloading curves under different strains at certain resting time (30min) between two successive measurements

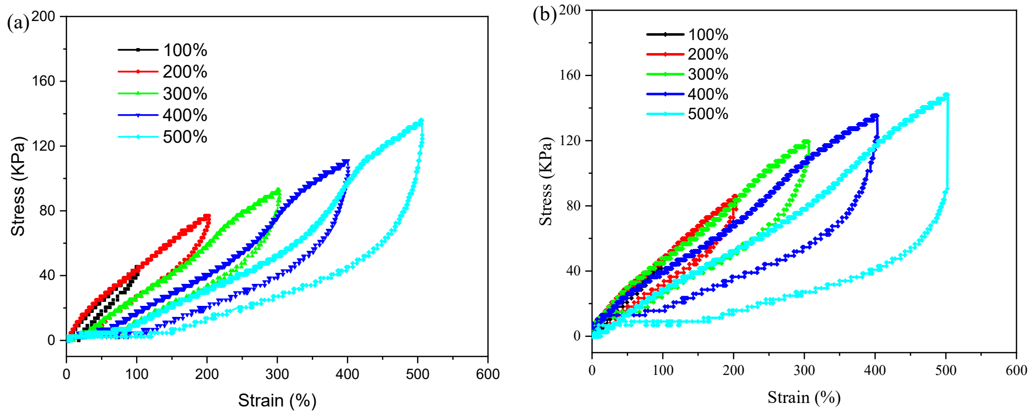

Figure S1 Hysteresis and self-recovery properties of P(AAc-co-AM)/PVA DN hydrogels at room temperature: (a) cyclic tensile loading-unloading curves under different strains at certain resting time (5min) between two successive measurements; (b) cyclic tensile loading-unloading curves under different strains at certain resting time (30min) between two successive measurements
